# *QuickStats*: Percentage* of Adults Aged ≥18 Years Who Took Prescription Medication During the Past 12 Months,^†^ by Sex and Age Group — National Health Interview Survey, United States, 2021^§^

**DOI:** 10.15585/mmwr.mm7216a7

**Published:** 2023-04-21

**Figure:**
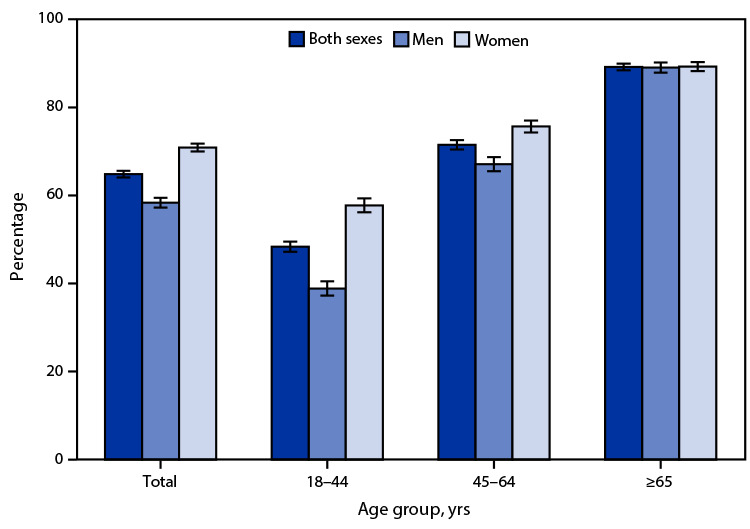
In 2021, 64.8% of adults aged ≥18 years took prescription medication at any time during the past 12 months. The percentage of adults taking prescription medication was lower among men than women overall (58.4% versus 70.9%) and for those aged 18–44 years (38.9% versus 57.8%) and 45–64 years (67.1% versus 75.7%). Among adults aged ≥65 years, men (89.0%) and women (89.3%) were equally likely to take prescription medication. Prescription medication use increased with age, from 48.4% for those aged 18–44 years to 89.2% for those aged ≥65 years, and this pattern of increasing use with age was observed for both men and women.

**Source:** National Center for Health Statistics; National Health Interview Survey, 2021. https://www.cdc.gov/nchs/nhis.htm

